# Trauma Sciences MSc: which students benefit from a postgraduate trauma degree?

**DOI:** 10.1186/1757-7241-21-S1-S14

**Published:** 2013-05-28

**Authors:** H Nnajiuba, H Rafique, S Adbul Latheef, D Neptune, A Guerrero

**Affiliations:** 1 Queen Mary University, London, UK

## Background

The Masters in Trauma Sciences at Queen Mary, University of London, is a unique didactic post-graduate degree, open to clinical personnel of varied specialty and experience.

Our primary aim was to measure whether there was any benefit from course participation. The secondary aims were to identify any differences in benefit based on level of training and specialty and to determine the perceived impact on future careers.

## Methods

A questionnaire was distributed to all inaugural students at the end of Year 1. Ten point scales, were used to enquire about pre and post-course benefit.

Benefit in this study was defined as an increase in knowledge and clinical confidence. Clinical confidence was defined as assurance in the care of trauma patients, journal interpretation and potential for being a leader in the field of trauma care. Comparisons were made between student groups based on level of training and specialty. Student aims were assessed by ‘yes/no’ enquiries about trauma career planning.

## Results

As this was the first year only 21 questionnaires were distributed. Twenty were returned (11 female; 9 male; mean ages 39.8±10.5 and 31.6±5.0 respectively). There were 5 consultants, 10 middle-grades, 2 junior doctors and 3 nurses. Nine had a general or trauma surgery background, 4 were from emergency medicine, 4 from anaesthesia and 3 from other clinical areas.

Overall, the pre-course and post-course mean knowledge scores were 5.15 and 7.95 respectively (+2.8). Pre and post-course mean scores for the consultant group were 6.8 vs 8.8 (+2); registrar group 3.5 vs 6.83 (+3.33); senior house officer group 4.6 vs 8 (+3.4) and house officer group 2 vs 8 (+6). According to specialty, pre and post-course mean scores were 4.33 vs 7 (+2.67) for emergency medicine, 5 vs 8.22 (+3.22) for surgery, 5 vs 8 (+3) for anaesthesia, 2 vs 7 (+5) for the military group and 7.67 vs 8.33 (0.66) for the nurses.

The class post-course confidence scores in trauma care provision, trauma journal interpretation and potential for being a leader in the field of trauma care were 8, 7.45 and 7.6 respectively. Mean post-course confidence scores in the same domains are given in the tables according level of training (table 1) and specialty (table 2).

**Table 1:** Post-course confidence scores based on level of training

	Provision of trauma care	Journal interpretation	Potential for being a trauma leader
**Consultant**	8.6	7.8	8.6

**Registrar**	7.67	7	6.17

**Senior house officer**	7.67	7.2	7.6

**House officer**	8	9	9

**Table 2:** Post-course confidence scores based on specialty

	Provision of trauma care	Journal interpretation	Potential for being a trauma leader
**Emergency medicine**	7.67	6.76	4.67

**Surgery**	7.78	7.56	8.11

**Anaesthesia**	8.5	8	8.5

**Military**	8	6	6

**Nurses**	8.33	7.67	8.33

With respect to student aims, 85% planned to be more active in a career in trauma compared to before the degree, 80% considered the degree to be a major career milestone and 75% were more likely to join a hospital committee.

## Conclusion

Participants benefited from this course. All groups had a similar increase in perceived knowledge, and a similar confidence after one year on the degree thus demonstrating that clinicians with diverse experience levels and backgrounds benefit uniformly. Furthermore, most considered it a major milestone in their career that would enhance their credentials. The authors are confident that cumulative data collection from the ensuing years of students will further support the growth of this course and other similar courses.

